# MethylStar: A fast and robust pre-processing pipeline for bulk or single-cell whole-genome bisulfite sequencing data

**DOI:** 10.1186/s12864-020-06886-3

**Published:** 2020-07-13

**Authors:** Yadollah Shahryary, Rashmi R. Hazarika, Frank Johannes

**Affiliations:** 1 Technical University of Munich, Institute for Advanced Study (IAS), Lichtenbergstr. 2a, Garching, 85748 Germany; 2 Technical University of Munich, Department of Plant Sciences, Liesel-Beckmann-Str. 2, Freising, 85354 Germany

**Keywords:** DNA methylation, Whole genome bisulfite sequencing, NGS, Pipeline, Single cell

## Abstract

**Background:**

Whole-Genome Bisulfite Sequencing (WGBS) is a Next Generation Sequencing (NGS) technique for measuring DNA methylation at base resolution. Continuing drops in sequencing costs are beginning to enable high-throughput surveys of DNA methylation in large samples of individuals and/or single cells. These surveys can easily generate hundreds or even thousands of WGBS datasets in a single study. The efficient pre-processing of these large amounts of data poses major computational challenges and creates unnecessary bottlenecks for downstream analysis and biological interpretation.

**Results:**

To offer an efficient analysis solution, we present MethylStar, a fast, stable and flexible pre-processing pipeline for WGBS data. MethylStar integrates well-established tools for read trimming, alignment and methylation state calling in a highly parallelized environment, manages computational resources and performs automatic error detection. MethylStar offers easy installation through a dockerized container with all preloaded dependencies and also features a user-friendly interface designed for experts/non-experts. Application of MethylStar to WGBS from Human, Maize and *A. thaliana* shows favorable performance in terms of speed and memory requirements compared with existing pipelines.

**Conclusions:**

MethylStar is a fast, stable and flexible pipeline for high-throughput pre-processing of bulk or single-cell WGBS data. Its easy installation and user-friendly interface should make it a useful resource for the wider epigenomics community. MethylStar is distributed under GPL-3.0 license and source code is publicly available for download from github https://github.com/jlab-code/MethylStar. Installation through a docker image is available from http://jlabdata.org/methylstar.tar.gz

## Background

Whole-Genome Bisulfite Sequencing (WGBS) is a Next Generation Sequencing (NGS) technique for measuring DNA methylation at base resolution. As a result of continuing drops in sequencing costs, an increasing number of laboratories and international consortia (e.g. IHEC, SYSCID, BLUEPRINT, EpiDiverse, NIH ROADMAP, Arabidopsis 1001 Epigenomes, Genomes and physical Maps) are adopting WGBS as the method of choice to survey DNA methylation in large population samples or in collections of cell lines and tissue types, either in bulk or at the single-cell level [1, 2]. Such surveys can easily generate hundreds or even thousands of WGBS datasets in a single study. A broad array of software solutions for the downstream analysis of bulk and single-cell WGBS data have been developed in recent years. These include tools for data normalization (e.g. RnBeads [3], SWAN [4], ChAMP [5]), detection of differentially methylated regions (DMRs) (e.g. Methylkit [6], DMRcaller [7], Methylpy [8], metilene [9]), imputation of methylomes from bulk WGBS data (e.g. METHimpute [10]), imputation of single-cell methylomes (e.g. Melissa [11], deepCpG [12]) and dropouts in single-cell data (e.g. SCRABBLE [13]).

**Fig. 1:**
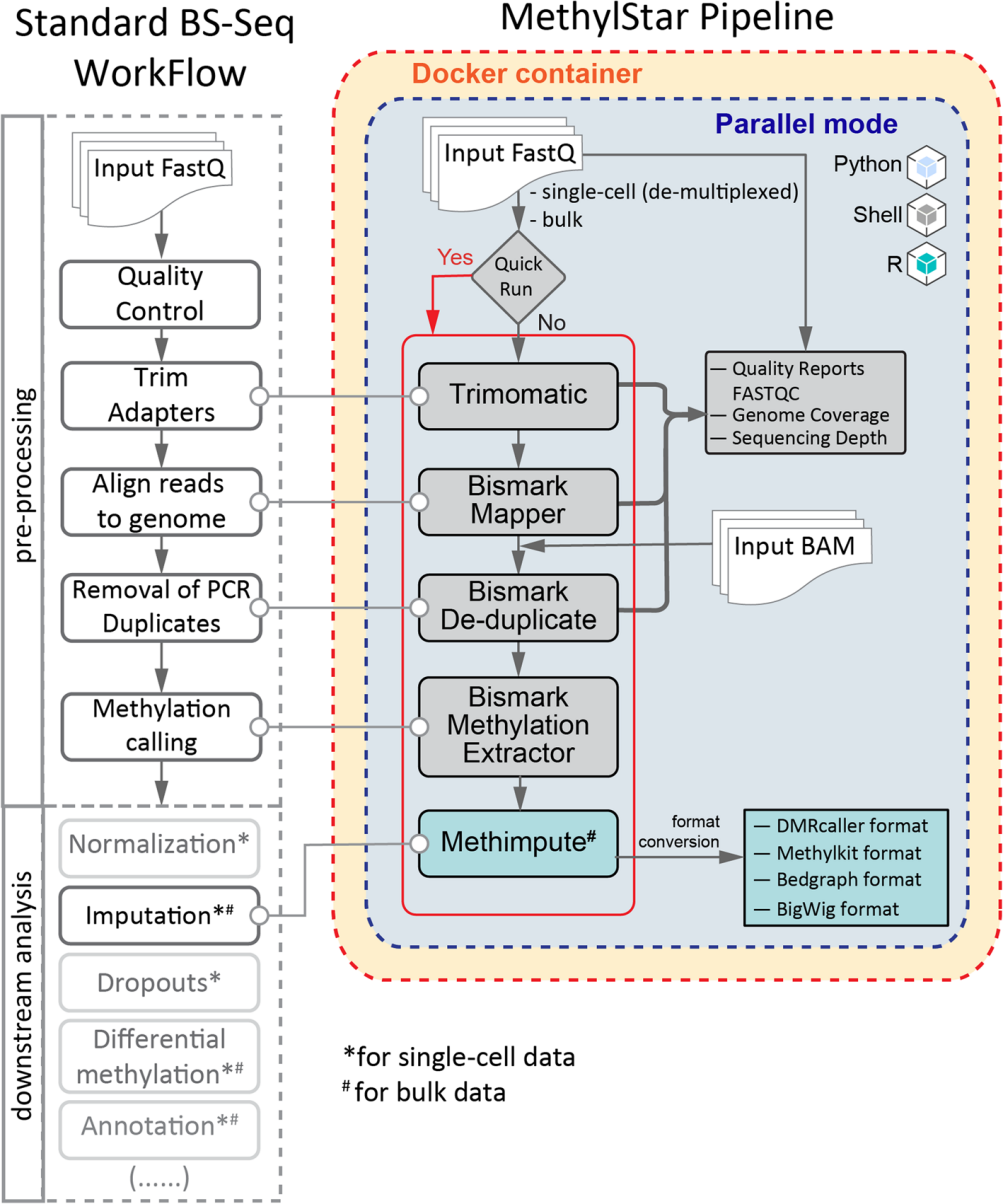
Basic workflow of MethylStar showing the pipeline architecture. The left panel shows a standard BS-Seq workflow and on the right are the different components of the MethylStar pipeline integrated as 3 different layers viz. Python, Shell and R. All steps of the pipeline have been parallelized using GNU parallel. MethylStar offers the option for “Quick run” (indicated in red) which runs all steps sequentially in one go or each component can be executed separately. MethylStar incorporates all pre-processing steps of a standard BS-Seq workflow and generates standard outputs that can be used for input into several downstream analysis tools

However, these downstream analysis tools are dependent on the output of a number of data pre-processing steps, such as quality control (e.g. FastQC (https://www.bioinformatics.babraham.ac.uk/projects/fastqc), QualiMap [14], NGS QC toolkit [15]), de-multiplexing of sequence reads, adapter trimming (e.g. Trimmomatic [16], TrimGalore (https://github.com/FelixKrueger/TrimGalore), Cutadapt [17]), alignment of reads to a reference genome and generation of methylation calls (e.g. BSseeker2 [18], BSseeker3 [19], Bismark [20], BSMap [21], bwa-meth (https://github.com/brentp/bwa-meth/), BRAT-nova [22], BiSpark [23], WALT [24], segemehl [25]). From a computational standpoint, data pre-processing is by far the most time-consuming step in the entire bulk or single-cell WGBS analysis workflow (Fig.1). In an effort to help streamline the pre-processing of WGBS data several pipelines have been published in recent years. These include nf-core/methylseq [26], gemBS [27], Bicycle [28] and Methylpy, some of which are currently employed by several epigenetic consortia. gemBS, Bicycle and Methylpy integrate data pre-processing and analysis steps using their own custom trimming and/or alignment tools (see Table 1). By contrast, nf-core/methylseq implements well-established NGS tools, such as TrimGalore for read trimming and Bismark and bwa-meth/MethylDackel for alignment. The nf-core/methylseq framework is built using Nextflow [29], and aims to provide reproducible pipeline templates that can be easily adapted by both developers as well as experimentalists. Despite these efforts, the installation and execution of these pipelines is not trivial and often require substantial bioinformatic support. Moreover, managing the run times of these pipelines for large numbers of WGBS datasets (i.e. in the order of hundreds or thousands) relies on substantial manual input, such as launching of parallel jobs on a compute cluster and collecting output files from temporary folders.

**Table 1:** Table showing different features of MethylStar as compared to other BS-seq pipelines

	Methylpy	MethylStar	methylseq	gemBS	Bicycle
Pipeline Features					
Multi-threading		*√*	*√*	*√*	*√*
language	Python	Python, shell, R	Java	C, Python	Java
distribution	github, PyPI	GitHub	Github	GitHub	Github
	(Apache license)	(GNU GPL3)	(MIT license)	(GNU GPL3)	(GNU GPL3)
Installation &	pip install, install	Docker, install	Docker,	Docker,	Docker
configuration	dependencies	dependencies	Singularity,	Singularity	
			Conda		
User-interface	-	*√*	-	-	-
Single/paired-end	*√*	*√*	*√*	*√*	*√*
Input data	Single-cell, WGBS,	WGBS, Single-cell	WGBS	RRBS, WGBS,	WGBS
	singlecell NOMe-seq, PBAT	(PBAT)		PBAT	
Pipe steps					
adapter trimming	Cutadapt	Trimmomatic	TrimGalore	-	bicycle analyzemethylation
alignment	bowtie/bowtie2	Bismark	Bismark,	gem3	bicycle align/
			bwa-meth		bowtie/bowtie2
remove PCR	Picard	Bismark	Bismark, Picard	Bscall	bicycle analyzemethylation
duplicates					
methylation	*√*	ProcessBismarAln,	Bismark,	Bscall	bicycle
calling		Bismark	MethylDackel		analyzemethylation, GATK
imputation of	-	METHimpute	-	-	-
missing cytosines					
DMR calling	*√*	-	-	-	bicycle analyze
					differential
					methylation
SNP calling	-	-	-	Bscall	-
Alignment QC	-	Bismark	Qualimap	*√*	*√*
summary reports	*√*	FastQC	Bismark,	*√*	*√*
			MultiQC, Preseq		
Methylation	BigWig	BigWig, bedGraph	-	BigWig,	BigWig
visualization				bedGraph	

In an attempt to address these issues, we have developed MethylStar, a fast, stable and flexible pre-processing pipeline for WGBS data. MethylStar integrates well-established NGS tools for read trimming, alignment and methylation state calling in a highly parallelized environment, manages computational resources and performs automatic error detection. MethylStar offers easy installation through a dockerized container with all preloaded dependencies and also features a user-friendly interface designed for experts/non-experts. Application of MethylStar to WGBS from Human, Maize and *A. thaliana* shows favorable performance in terms of speed and memory requirements compared with existing pipelines.

## Implementation

### Core pipeline NGS components

In its current implementation, MethylStar integrates processing of raw fastq reads for both single- and paired-end data with options for adapter trimming, quality control (fastQC) and removal of PCR duplicates (Bismark software suite). Read alignment and cytosine context extraction is performed with the Bismark software suite. Alignments can be performed for WGBS and Post-bisulfite adaptor tagging (PBAT) approaches for single-cell libraries. Bismark was chosen because it features one of the most sensitive aligners, resulting in comparatively high mapping efficiency, low mapping bias and good genomic coverage [30, 31]. Finally, cytosine-level methylation calls are (optionally) obtained with METHimpute, a Hidden Markov Model for inferring the methylation status/level of individual cytosines, even in the presence of low sequencing depth and/or missing data. All the different data processing steps have been optimized for speed and performance (see below), and can run on local machines as well as on larger compute nodes.

### User interface

MethylStar features a lightweight python-based user interface, which is particularly useful for bench-scientists who are not familiar with command-line scripting. The aim of the interface is to improve useability and to reduce human error arising from typing mistakes or from the misspecification of parameter settings during pipeline configuration. The interface offers configuration templates that can be easily re-used for subsequent samples/projects, thus ensuring consistency and repeatability of data analysis projects. Unlike many web-based or graphical-based interfaces, the MethylStar interface does not require additional resources and/or dependencies. Users navigate through an index menu and run selected pipeline components by typing the menu index of choice. We designed the interface for both experts and non-experts. Non-experts are able to execute all pipeline commands without having to edit a single bash script, while advanced users can easily configure additional parameters and install software/tools (e.g. most recent/legacy version of a software) to integrate with MethylStar by simply specifying path variables. Finally, users can configure email addresses to receive automatic notifications when a job completed or failed. A video demonstrating the use of the interface can be found at https://github.com/jlab-code/MethylStar#MethylStar_tutorial_on_YouTube.

### Pipeline architecture, optimization of parallel processes and memory usage

The pipeline architecture comprises three main layers (Fig. 1). The first layer is the interactive command-line user interface implemented in Python to simplify the process of configuring software settings and running MethylStar. The second layer consists of shell scripts, and handles low-level processes, efficiently coordinates the major software components and manages computational resources. The final layer is implemented in R, and is used to call METHimpute and to generate output files that are compatible with a number of publicly available DMR-callers such as Methylkit, DMRcaller and bigWig files for visualization in Genome Browsers such as JBrowse [32]. All outputs are provided in standard data formats for downstream analysis.

All components/steps of the pipeline have been parallelized using GNU Parallel (https://www.gnu.org/software/parallel/) (Fig. 1). The user can either set the number of parallel jobs manually for each pipeline component, or can opt to use the inbuilt parallel option from the “configuration” option of the menu. The inbuilt parallel implementation is also available under the “Quick Run” option. This latter option detects the number of parallel processes/jobs automatically for each pipeline component based on available system cores/threads and memory, thus allowing the user to run the entire steps of the pipeline in one go.

In the parallel implementation of all pipeline steps, we use genome size (in base pairs) as an additional factor in the optimization of computational resources. For example, in the analysis of *A. thaliana* samples (genome size ∼135 mega base pairs), our parallel implementation of Trimmomatic (a java tool) sets the optimal number of jobs to 12 on a system with 88 cores and 386 GB RAM. This setting allocates (12 jobs ×8 threads) =96 threads for trimming (java threads) and (12 jobs ×1 threads) =12 threads to the gzip tools (default no. of threads fixed to 8 in the pipeline). By contrast, for read trimming in Maize (genome size ∼2500 mega base pairs), the optimal number of jobs is set to 5. In the parallel implementation of Bismark alignment step under a similar system configuration, while running paired-end reads from *A. thaliana*, we optimally set the number of jobs to 4. This setting allocates (4 jobs ×8 files/threads) =32 threads to Bowtie2 and (4 jobs ×8 files/threads ×2) =64 threads to the bismark alignment tool (default no. of threads fixed to 8 in the internal bismark parallel argument). In a similar way, for deduplicate_bismark, the optimal number of jobs is set to (1/4th of total 88 cores) =22. For bismark_methylation_extractor it is set as 4, which allocates (4 jobs ×8 threads) =32 threads each to itself and to Bowtie tools as well as a few additional cores to gzip and samtools streams. In this way, the maximum number of threads never exceeds the total number of available cores, which in turn allows other jobs such as file compression, I/O operations to be performed simultaneously. Under the “Quick Run” option we have parallelized R processes such as the extraction of methylation calls from BAM files (post PCR duplicates removal) by bypassing the Bismark methylation extractor step and by passing these calls directly onto METHimpute for imputation of missing cytosines (Fig. 1).

### Automatic error handling and detection

MethylStar issues user-friendly messages related to configuration errors such as non-existing paths to input/output folders, low disk space, incorrect file extensions, non-empty folders. In addition, we have introduced checkpoints for each individual component of the pipeline so that a job can be resumed easily from the nearest checkpoint in the unlikely event of system failure (e.g. disk issues, file corruption, user interruption). MethylStar accepts intermediate files such as BAM files, CX-reports etc., and is able to process these new files together with pre-existing files in the folder. MethylStar issues user-friendly warnings before resuming each run. For instance, if a given folder is non-empty it will ask for user permission to continue, and issues a message that files with pre-existing names will be overwritten.

### Running MethylStar

The user can choose to run each pipeline component individually, and customize software settings at each step by editing the configuration file, which is available as an option through the interactive command-line user interface. The user interface displays the available options as an index menu, and users can execute specific pipeline steps. Some of the key configuration parameters include setting file paths to input and output data, options for handling large batches of samples, file format conversions, as well as options for deleting auxiliary files that are generated during intermediate analysis steps. Our interactive user interface aids in the fast execution of complex commands and will be particularly effective for users who are less familiar with command line scripting. As an alternative, MethylStar also features a “Quick Run option”, which allows the user to run all pipeline steps in one go using default configuration settings (Fig. 1).

### Installation and documentation

MethylStar can be easily installed via a Docker image. This includes all the softwares, libraries and packages within the container, and thus solves any dependency issues. Advanced users can edit the existing docker container and build their own image.

Detailed description about installation and running the pipeline is available at https://github.com/jlab-code/MethylStar.

## Results and discussion

### Benchmarking of speed

**Fig. 2:**
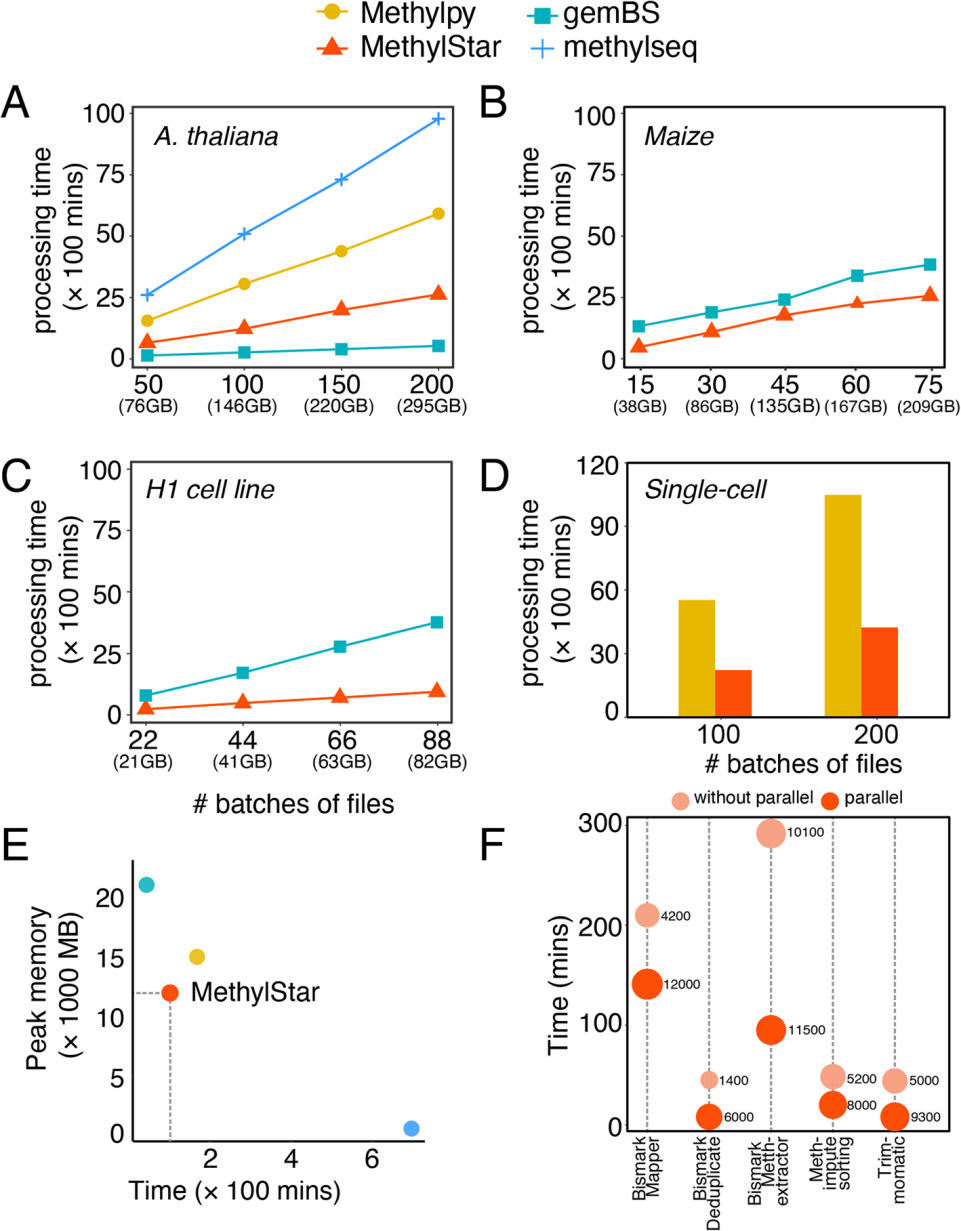
Performance of MethylStar as compared with other BS-Seq analysis pipelines viz. Methylpy, nf-core/methylseq and gemBS in (**a**) *A. thaliana* (**b**) Maize (**c**) H1 cell line and (**d**) scBS-Seq samples. CPU processing time taken by METHimpute was not included in the current benchmarking process as there is no equivalent method in the other pipelines to compare with. Because of the very long run times observed for the *A. thaliana* data, Methylpy and Methylseq were no longer considered for benchmarking of speed in Maize and H1 cell line samples. All pipelines were run using 32 jobs. (**e**) Peak memory usage as a function of time for 10 random *A. thaliana* samples. (**f**) Time taken by each component of MethylStar. X-axis shows the individual components of MethylStar where the dot with lighter shade of orange indicates -without parallel and darker shade of orange indicates - with parallel implementation of MethylStar. On the y-axis is the time in mins. The size of the dot indicates the peak memory usage in MB by each component

To demonstrate MethylStar’s performance we analyzed bulk WGBS data from a selection of 200 *A. thaliana* ecotypes (paired-end, 295 GB, ∼8.63× depth, 85.66% genome coverage, GSE54292), 75 Maize strains (paired-end, 209 GB, ∼0.36× depth, ∼22.12% genome coverage, GSE39232) and 88 Human H1 cell lines (single-end, 82 GB, ∼0.12× depth, ∼10.62% genome coverage, GSM429321). MethylStar was compared with Methylpy, nf-core/methylseq and gemBS. All pipelines were run with default parameters on a computing cluster with a total of 88 cores (CPU 2.2 GHz with 378 GB RAM). Speed performance was assessed for a series of batch sizes (*A. thaliana*: 50, 100, 150, 200 samples; Human H1 cell line: 22, 44, 66, 88 samples; Maize: 15, 30, 45, 60, 75 samples) and was restricted to a fixed number of jobs (=32), (Fig. 2a-c and Additional file 1: Table S2). Although gemBS achieved the fastest processing times for the *A. thaliana* samples, MethylStar clearly outperformed the other pipelines when applied to the more complex genomes of Maize and Human, which are computationally more expansive and resource-demanding (Fig. 2b-c). For instance, for 88 Human WGBS samples (82 GB of data), MethylStar showed a 75.61% reduction in processing time relative to gemBS, the second fastest pipeline (∼909 mins vs. ∼3727 mins). Extrapolating from these numbers, we expect that for 1000 Human WGBS samples, MethylStar could save about ∼22.24 days of run time (4 × faster). To show that MethylStar can also be applied to single-cell WGBS data, we analyzed DNA methylation of 200 single cells from Human early embryo tissue (paired-end, 845 GB, ∼0.38× depth, ∼9.97% genome coverage, GSE81233) split into batches of 100 and 200 (Fig. 2d and Additional file 1: Table S2). MethylStar’s processing times were compared to Methylpy which also supports single-cell data. For 100 cells, MethylStar required only ∼2225 mins as compared to ∼5518 mins required by Methylpy. Hence, MethylStar presents an efficient analysis solution for deep single-cell WGBS experiments.

To demonstrate that MethylStar’s processing speed does not come at the expense of poor read alignments, we analysed the read mapping statistics of 50 samples each of *A. thaliana*, Maize, Human H1 cell line and single-cell Human data using MethylStar, Methylpy, nf-core/methylseq and gemBS. Our results show that MethylStar and nf-core/methylseq, both of which employ the Bismark alignment tool, provide the most accurate and sensitive alignments. This observation that is consistent with recent benchmarking results [30, 31]. By contrast, Methylpy and gemBS use their own inbuilt aligners and generally display poorer alignment statistics. Interestingly, although gemBS was the fastest pipeline for the *A. thaliana* samples, the percentage of ambiguously mapped reads was considerably higher than that of MethylStar, thus demonstrating a trade-off between speed and mapping performance. We also noticed that the percentage of ambiguously mapped reads by gemBS was even further increased in the case of the Maize samples (Additional file 1: Fig. S1 and Table S1). This could indicate that gemBS’s alignment performance is particularly challenged in complex plant genomes, although this hypothesis should be explored in more detail.

### Memory usage statistics

Along with benchmarking of speed, we also evaluated the performance of the MethylStar, gemBS, nf-core/methylseq and Methylpy pipelines in terms of system memory utilization using the MemoryProfiler (https://github.com/pythonprofilers/memory_profiler) python module (Fig. 2e). We assessed the CPU time versus peak/max memory of all the 4 pipelines (default settings) on a computing cluster (specifications above). For 10 random samples from the above *A. thaliana* benchmarking dataset (paired-end, 16 GB, GSE54292) MethylStar and Methylpy showed the best balance between peak memory usage (∼12000 MB and ∼15000 MB, respectively) and total run time (∼177 mins and ∼333 mins, respectively). In contrast, nf-core/methylseq and gemBS exhibited strong trade-offs between memory usage and speed, with nf-core/methylseq showing the lowest peak memory usage (∼700 MB) but the longest CPU time (∼697 mins), and gemBS the highest peak memory usage (∼21000 MB) but the shortest run time (∼42 mins) (Fig. 2e and Additional file 1: Table S5).

Furthermore, we inspected the run times of MethylStar’s individual pipeline components, both with and without parallel implementation (Fig. 2f and Additional file 1: Table S3). Our results clearly show that the parallel implementation is considerably faster for all components; however, it is accompanied by a higher peak memory usage. For instance, the implementation of the Bismark alignment step required ∼141 mins (with parallel) as compared to ∼210 mins (without parallel), a ∼33% reduction in processing time. However, in exchange, peak memory usage was increased by ∼65%. Thus, with sufficient computational resources, MethylStar’s parallel implementation of Bismark alignment can be very effective in handling large numbers of read alignments in considerably less amount of time (Fig. 2f).

We further benchmarked memory usage using 10 random samples from the above Maize dataset (paired-end, 23 GB, GSE39232). For this analysis, we focused on gemBS and MethylStar due to their shorter processing times for these datasets as compared to nf-core/methylseq and Methylpy. For these Maize dataset, gemBS’s peak memory usage was ∼110000 MB as compared to ∼81000 MB for MethylStar (∼1.3 times less memory), (Additional file 1: Table S4) with a total run time of ∼667 mins and ∼508 mins, respectively. We observed a 76% reduction in processing times of Maize samples using the parallel implementation of MethylStar pipeline (Additional file 1: Table S4) as compared to the without parallel implementation. Taken together, these benchmarking results clearly show that MethylStar exhibits favorable performance in terms of processing time and memory, and that it is therefore an efficient solution for the pre-processing of large numbers of samples even on a computing cluster with limited resources.

## Conclusion

MethylStar is a fast, stable and flexible pipeline for the high-throughput analysis of bulk or single-cell WGBS data. Its easy installation and user-friendly interface should make it a useful resource for the wider epigenomics community.

## Availability and requirements

Project name: MethylStarProject home page: https://github.com/jlab-code/MethylStarOperating system(s): Cross-platformProgramming language: Python, Shell, R License: GPL-3.0

## Supplementary information

**Additional file 1** Supplementary figures and data tables (pdf format) showing mapping statistics, processing times and memory usage of different pipelines benchmarked.

## Data Availability

Not applicable

## Abbreviations

WGBS: Whole-genome bisulfite sequencing
NGS: Next generation sequencing
DMRs: Differentially methylated regions
QC: Quality control
PCR: Polymerase chain reaction
PBAT: Post-bisulfite adaptor tagging
CX-reports: Cytosine context (CG, CHG, CHH) report for all cytosines
BAM: Binary alignment map
RAM: Random-access memory
CPU: Central processing unit
MB: Mega bytes
GB: Giga bytes
I/O: Input/output
