# Supplementary material for: MethylStar: A fast and robust pre-processing pipeline for bulk or single-cell whole-genome bisulfite sequencing data
Source: BMC Genomics. 2020 Jul 13;21:479. doi: 10.1186/s12864-020-06886-3 (PMC7359584; doi:10.1186/s12864-020-06886-3)
Supplement: Supplementary file 1 — Additional file 1 Supplementary figures and data tables (pdf format) showing mapping statistics, processing times and memory usage of different pipelines benchmarked. [file 12864_2020_6886_MOESM1_ESM.pdf]

Supplementary Figure 1

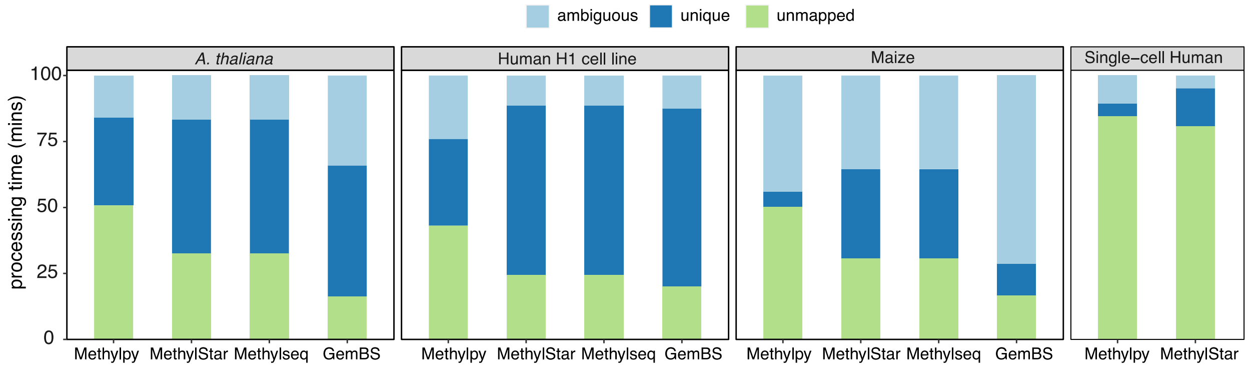

Figure S1. Read mapping statistics of each pipeline run with 50 samples showing percentage of ambiguously, uniquely, and unmapped reads. The values are shown in Supplementary Table 1.

Supplementary Table 1

	Methylpy	MethylStar	methylseq	gemBS
**Overall alignment rate (%)**				
A. thaliana	49.19	67.41	67.41	83.79
Maize	70.76	69.38	69.38	83.33
Human H1 cell line	56.90	75.57	75.57	79.95
Single-cell Human	15.38	19.21	-	-
**Uniquely mapped reads (%)**				
A. thaliana	33.30	50.67	50.67	49.70
Maize	7.35	33.91	33.91	11.98
Human H1 cell line	32.87	64.25	64.25	67.47
Single-cell Human	4.69	14.39	-	-
**Ambiguously mapped reads (%)**				
A. thaliana	15.89	16.74	16.74	34.09
Maize	63.41	35.47	35.47	71.35
Human H1 cell line	24.03	11.32	11.32	12.48
Single-cell Human	10.69	4.82	-	-
**Unmapped reads (%)**				
A. thaliana	50.81	32.59	32.59	16.21
Maize	29.24	30.62	30.62	16.67
Human H1 cell line	43.10	24.43	24.43	20.05
Single-cell Human	84.62	80.79	-	-

Table S1. Read mapping statistics of each pipeline run with 50 samples. Overall alignment rate is the total of uniquely and ambiguously mapped reads.

Supplementary Table 2

species	# batches	Methylpy	MethylStar	methylseq	gemBS
A. thaliana	50	1555	656	2603	136
A. thaliana	100	3055	1230	5090	264
A. thaliana	150	4386	1995	7305	396
A. thaliana	200	5916	2626	9778	529
Maize	15	-	475	-	1329
Maize	30	-	1096	-	1897
Maize	45	-	1779	-	2415
Maize	60	-	2252	-	3386
Maize	75	-	2573	-	3842
Human H1 cell line	22	-	202	-	760
Human H1 cell line	44	-	451	-	1683
Human H1 cell line	66	-	673	-	2746
Human H1 cell line	88	-	909	-	3727
Single-cell Human	100	5518	2225	-	-
Single-cell Human	200	10482	4227	-	-

Table S2. Processing times of each pipeline in minutes.

Supplementary Table 3

	Parallel		without Parallel	
**MethylStar components**	**Time (mins)**	**Memory (MB)**	**Time (mins)**	**Memory (MB)**
Trimmomatic	8	9300	44	5000
Bismark Mapper	141	12000	210	4200
deduplicate_bismark	8	6000	45	1400
bismark_methylation_extractor	95	11500	294	10100
Methimpute sorting	20	8000	47	5200

Table S3. Processing time and peak memory usage of different components of MethylStar using 10 samples of *A. thaliana*

Supplementary Table 4

	Parallel		without Parallel	
**MethylStar components**	**Time (mins)**	**Memory (MB)**	**Time (mins)**	**Memory (MB)**
Trimmomatic	20	8000	92	1300
Bismark Mapper	254	81000	1417	14000
deduplicate_bismark	8	4100	23	1600
bismark_methylation_extractor	195	30000	507	17500
Methimpute sorting	31	30000	66	25000

Table S4. Processing time and peak memory usage of different components of MethylStar using 10 samples of Maize

Supplementary Table 5

Pipelines	Time (mins)	Memory (MB)
Methylpy	333	15000
MethylStar	177	12000
methylSeq	697	700
gemBS	42	21000

Table S5. Processing time and peak memory usage of each pipeline using 10 samples of *A. thaliana.* MethylStar was run using the "Quick Run" option where methylation calls were directly extracted from BAM files using ProcessBismarkAln function from Methylkit.
